# Micro-CT Evaluation of Microgaps at Implant–Abutment Connection

**DOI:** 10.3390/ma16124491

**Published:** 2023-06-20

**Authors:** Jakub Kowalski, Adam K. Puszkarz, Mateusz Radwanski, Jerzy Sokolowski, Michal Cichomski, Rim Bourgi, Louis Hardan, Salvatore Sauro, Monika Lukomska-Szymanska

**Affiliations:** 1 Department of General Dentistry, Medical University of Lodz, 92-213 Lodz, Poland; 2 Faculty of Material Technologies and Textile Design, Institute of Material Science of Textiles and Polymer Composites, Lodz University of Technology, 116 Zeromskiego Street, 90-924 Lodz, Poland; 3 Department of Endodontics, Medical University of Lodz, 92-213 Lodz, Poland; 4 Department of Material Technology and Chemistry, Faculty of Chemistry, University of Lodz, Pomorska 163, 90-236 Lodz, Poland; 5 Department of Restorative Dentistry, School of Dentistry, Saint-Joseph University, Beirut 1107 2180, Lebanon; 6 Dental Biomaterials and Minimally Invasive Dentistry, Departamento de Odontología, Facultad de Cienciasde la Salud, Universidad CEU-Cardenal Herrera, C/Del Pozo ss/n, Alfara del Patriarca, 46115 Valencia, Spain; 7 Department of Therapeutic Dentistry, I. M. Sechenov First Moscow State Medical University, 119146 Moscow, Russia

**Keywords:** dental implant, microgap, micro-computed tomography, abutment

## Abstract

The assessment of microgaps at the implant–abutment interface is an important factor that may influence clinical success. Thus, the aim of this study was to evaluate the size of microgaps between prefabricated and customised abutments (Astra Tech, Dentsply, York, PA, USA; Apollo Implants Components, Pabianice, Poland) mounted on a standard implant. The measurement of the microgap was performed using micro-computed tomography (MCT). Due to 15-degree rotation of samples, 24 microsections were obtained. Scans were performed at four levels established at the interface between the abutment and the implant neck. Moreover, the volume of the microgap was evaluated. The size of the microgap at all measured levels varied from 0.1 to 3.7 µm for Astra and from 0.1 to 4.9 µm for Apollo (*p* > 0.05). Moreover, 90% of the Astra specimens and 70% of the Apollo specimens did not exhibit any microgaps. The highest mean values of microgap size for both groups were detected at the lowest portion of the abutment (*p* > 0.05). Additionally, the average microgap volume was greater for Apollo than for Astra (*p* > 0.05). It can be concluded that most samples did not exhibit any microgaps. Furthermore, the linear and volumetric dimensions of microgaps observed at the interface between Apollo or Astra abutments and Astra implants were comparable. Additionally, all tested components presented microgaps (if any) that were clinically acceptable. However, the microgap size of the Apollo abutment was higher and more variable than that of the Astra one.

## 1. Introduction

Dental implants allow us to both restore a single tooth and perform a full-arch prosthetic restoration. Single crowns, bridges or prostheses can be fixed on implants and significantly improve quality of life [1]. Implants are indicated in cases of edentulism, especially when adjacent teeth are healthy or when a removable denture is poorly tolerated by the patient [2,3].

Maintaining the bone level around the implant is the key issue in implant treatment. There are many factors influencing bone loss, such as systemic, social and local factors. The patient’s age, genetic predisposition and general conditions are related to systemic factors, while oral hygiene, stimulant consumption and socioeconomic factors are related to social ones [2]. The local characteristics comprise biological and mechanical factors, including the size of the microgap between the implant and the abutment [4]. The abutment and implant shape, the type of implant–abutment connection (external, internal, conical and their modifications), implant and abutment material, tightening torque value of the screw and marginal fit between components may influence the size of the microgap. This latter item can be defined as an open space at the implant–abutment interface [5].

The highest bone loss and largest microgaps are typically observed for the external implant–abutment connection [6] because the abutment is wider at the connection level than the implant. As a consequence, screw loosening and rotational misfit can lead to implant disintegration [4,6]. On the other hand, the development of an internal connection might reduce the bone loss and improve implant stability, thus enhancing the overall survival rate of implants [7]. Various geometries of internal connection have been introduced, namely internal hexagon, internal octagon, conical and tube-in-tube connection [4,8]. The load of the conical connection is transferred along two conical constructions, resulting in even distribution of forces. Additionally, it provides an ideal match between the implant and the abutment, resulting in isolation of the implant interior part from the oral cavity. As a consequence, microleakage and the risk of the abutment loosening quickly are significantly decreased [9]. Moreover, the optimal force distribution observed in tube-in-tube connection is the result of the construction, which is based on two cylinders. The longer the tube-in-tube connection, the better the durability of the implant system [10,11]. The internal connection, named platform switching, often exhibits better stability and less bone loss owing to horizontal offset. This modification was based on the implant shoulder, which is wider than an abutment, keeping it away from marginal bone surrounding the implant. As a result, less bone loss and plaque deposition around the implant–abutment connection are observed [12].

Moreover, the size of the microgap and the preservation of healthy soft tissues depend on the abutment material, its design, surface topography and preparation [13,14]. Nowadays, implants and abutments are mainly made of titanium (Ti) and its alloys [15]. The main alloy used in dental implantology is commercially pure titanium (CpTi) (grade IV) and Ti6Al4V (α-β combination) [16]. It should be emphasized that Ti alloys are considered as a gold standard in implantology due to their properties, such as thermal expansion similar to bone, relatively low Young’s modulus, low density, high strength, high corrosion resistance, very good biocompatibility and good osseointegration [17].

Apart from the above-mentioned items, the position of the implant–abutment connection relative to the alveolar crest may be responsible for inflammation around implants [18]. In this regard, implants may be divided into three types: (i) subcrestal, (ii) bone- and (iii) tissue-level. Subcrestally positioned implants may reduce the probability of implant exposure, therefore providing good aesthetics and reducing the risk of inflammation [18]. On the contrary, bone-level implants provide an adequate emergence profile around implants and appropriate soft-tissue adherence [19]. Tissue-level implants positioned above the level of the bone gained popularity due to lower bone loss and good hygiene around reconstructions [20].

Misfit of the implant–abutment interface can cause biological and mechanical failures. The former is associated with bacteria contamination and colonisation of the microgap between the implant and the abutment. During function, the movement of bacteria outside and inside of the implant–abutment interface (pumping effect) may occur. This phenomenon can lead to inflammation and tissue loss around the implant [21]. Moreover, the amount of bone loss can increase with the size of the implant–abutment microgap and colonisation of microorganisms [22]. It is worth emphasizing that mechanical failures related to implant–abutment misfit include loosening of prosthetic structure, abutment rotation, fracture of the abutment or abutment screw, bone microfractures or fracture of the implant body [23,24,25]. Additionally, the application of non-original abutments causes higher rotational misfit at the interface [26].

Various strategies were employed to measure the microgap at the interface between the implant and the abutment: sectioning and scanning electron microscopy (SEM) measurements, direct observation with optical microscope or micro-computed tomography (MCT) [27,28,29,30]. The sectioning and measurements using SEM led to irreversible damage of the specimen and therefore cannot be applied to compare objects pre- and post-load. On the other hand, direct observation cannot be performed when the implant–abutment connection is angled at different focal planes. Hence, the use of MCT is a very fast and precise measurement method providing evaluation up to the resolution of 0.5 µm [31,32]. Additionally, the high contrast ensures precise analysis, and MCT images are three-dimensional and support geometry analysis of samples. This non-invasive method might be used for precise length measurements and calculation of the area and volume of tested objects [31,33]. A further advantage of MCT is the ability to use ferromagnetic samples. However, the high cost can be considered as a major disadvantage [34]. Interestingly, there are no available studies establishing standards of microgap measurement.

The aim of the present study was to compare the microgap at the implant–abutment interface between two different implant–abutment systems. The null hypothesis was that there was a significant difference between the microgaps at the implant–abutment interfaces of the two different implant–abutment systems investigated.

## 2. Materials and Methods

### 2.1. Materials

**Figure 1:**
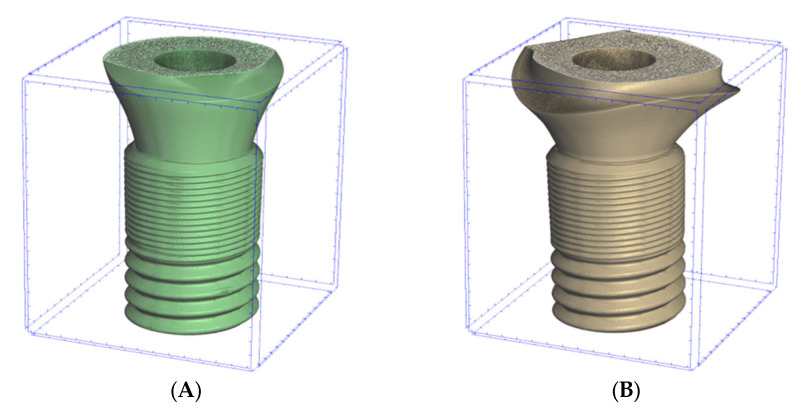
Sectioned three-dimension (3D) images of prepared samples: (**A**)—Astra; (**B**)—Apollo.

In this study, implant–abutments with conical internal connections were divided into two groups: Astra and Apollo. The first one was an implant OsseoSpeed EV (Astra Tech, Dentsply, York, PA, USA) and a prefabricated abutment TiDesign EV (Astra Tech, Dentsply, York, PA, USA). Apollo was an implant OsseoSpeed EV with a titanium customised abutment (Apollo Implants Components, Pabianice, Poland) (Table 1, Figure 1). Abutments were fixed to implants using the torque value (25 N·cm) recommended by the manufacturer.

The sample size was calculated using Statistica v. 13.1 (StatSoft, Inc., Tulsa, OK, USA) with the following parameters: effect size of 10%, standard deviation of 7%, significance level of 0.05 and power of 80%. The minimum sample size of 9 was determined. Thus, for each group, 10 specimens were prepared.

### 2.2. Microtomography Measurements

The specimens were placed in a special holder (Bruker, Kontich, Belgium) in MCT (Skyscan 1272; Bruker, Kontich, Belgium) under the following scanning conditions: X-ray source voltage, 90 kV; X-ray source current, 111 µA; and pixel size, 3.5 µm. The rotation step of 0.3° was applied, and aluminium (0.5 mm) + copper (0.038 mm) filters were selected. During the examination, the position of the specimens and the environment parameters (temperature, humidity) remained unchanged [35,36]. NRecon 1.7.4.2 and CTvox 3.3.0 r1403 software (Bruker, Kontich, Belgium) were used for 3D reconstruction of the implants. The measurements of anonymized samples were calculated using CTAn 1.17.7.2+ software (Bruker, Kontich, Belgium).

#### 2.2.1. Horizontal Microgap (Linear Measurement)

**Figure 2:**
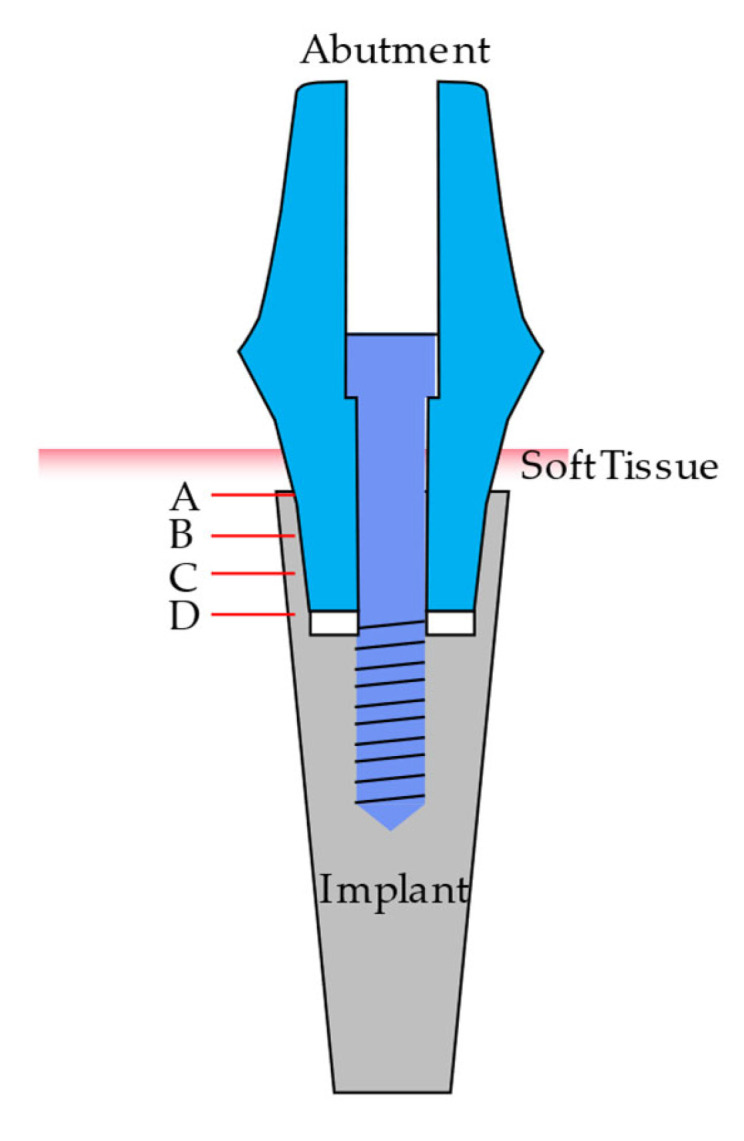
Implant–abutment interface. Microgap measurement levels (A–D).

The mating surface of the implant and abutment was measured at 4 levels (A, B, C, D) (Figure 2). Level A was defined at the neck of the implant, while D was defined at the lowest portion of the abutment. Levels B and C were positioned at equal distances ($\frac{1}{3}$ and $\frac{2}{3}$, respectively) between points A and D. Additionally, implants were rotated at 15 degrees, resulting in 24 sections for microgap detection and measurement [35]. 

#### 2.2.2. Volume of Microgap

Three-dimensional (3D) visualization was achieved using CTvol v2.3.2.0 software (Bruker, Kontich, Belgium). The selection of the region of interest relied on the contrast between various image areas. This phenomenon was caused by the un-like values of X-ray absorption for elements of scanned objects resulting from the difference in density, i.e., the X-ray absorption of the abutment and implant differed (was higher) from the X-ray absorption of air. As a consequence, evaluated elements of the scanned object could be easily selected [36,37].

### 2.3. Statistical Analysis

The normality of the data distribution was tested using the Shapiro–Wilk test. The U Mann–Whitney test was used for the comparison between the groups. The entire statistical analysis was performed using the statistical software package Statistica v. 13.1 (StatSoft, Inc., Tulsa, OK, USA), and statistical significance was considered at *p* < 0.05. A chi-square test was used to compare the qualitative variables.

## 3. Results

### 3.1. Horizontal Microgap (Linear Measurement)

**Figure 3:**
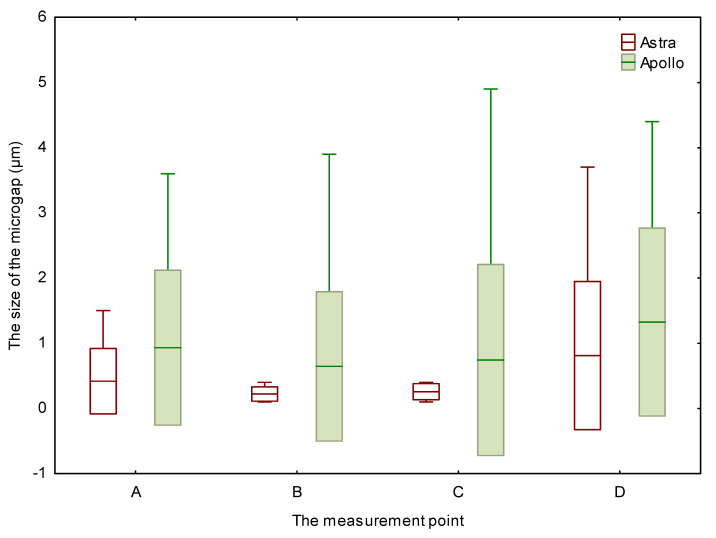
The size of the microgap at different levels for Apollo and Astra.

Firstly, 87.5% (70/80) of scans for Astra and Apollo did not present any microgaps (Appendix A, Table A1). Secondly, nine out of ten Astra specimens did not exhibit any microgaps, either. Moreover, for Apollo, a microgap was found in 30% (3/10) of the specimens; a greater scatter of results for Apollo was also observed (Figure 3). However, no significant difference between study groups was encountered.

The overall microgap size at all measured levels varied from 0.1 to 3.7 µm for Astra and from 0.1 to 4.9 µm for Apollo. Although the mean value of microgaps was larger for Apollo than for Astra, there was no significant difference between both groups.

Moreover, the smallest microgaps for Astra and Apollo were detected at levels B and C. At level A, the average value of microgaps reached 0.42 µm for Astra and 0.93 µm for Apollo. At level B, the microgap was equal to 0.22 µm for Astra and 0.65 µm for Apollo. Additionally, at level C, microgaps of 0.26 µm for Astra and 0.74 µm for Apollo were observed. The measurement at level D showed a microgap of 0.81 µm for Astra and 1.33 µm for Apollo. However, no significant differences between microgaps at different levels were encountered.

**Figure 4:**
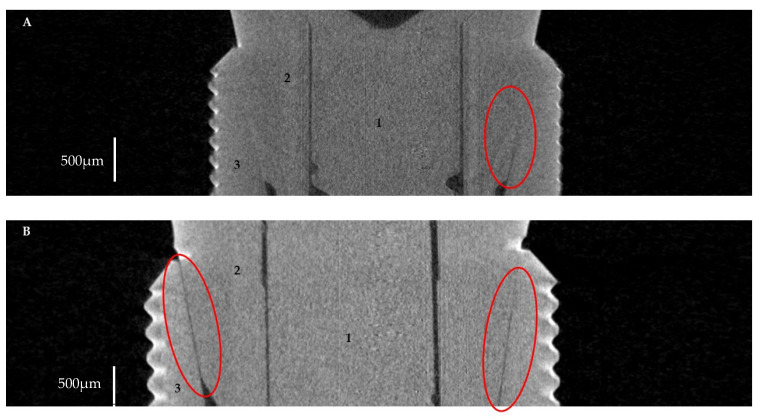
Implant–abutment interface with microgaps (red circle): (**A**)—Astra; (**B**)—Apollo. 1—screw, 2—abutment, 3—implant.

**Figure 5:**
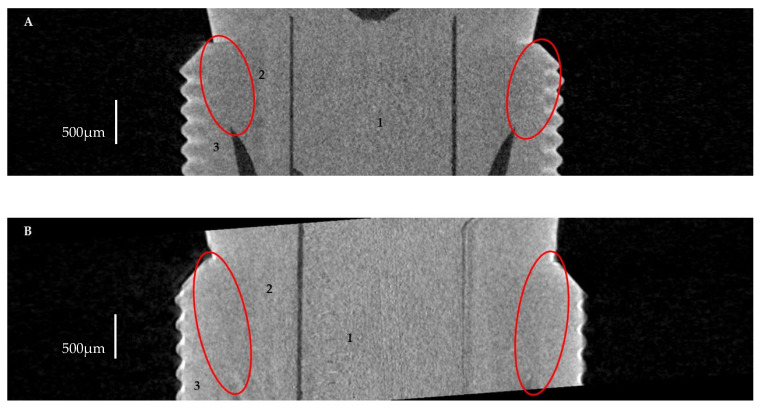
Implant–abutment interface without microgaps (red circle): (**A**)—Astra; (**B**)—Apollo. 1—screw, 2—abutment, 3—implant.

Figure 4 shows micro-CT images presenting microgaps between implants and abutments. In the case of Astra, the microgap was observed at level D, decreasing towards level C without microgaps at other levels (Figure 4A). In the case of Apollo, microgaps were visible at all measured levels of the implant–abutment connection (0.1 to 4.9 µm) (Figure 4B). However, in most of the specimens, microgaps were not found and an ideal fit between the implant and the abutment was detected (Figure 5A,B).

### 3.2. The Volume of Microgaps

**Figure 6:**
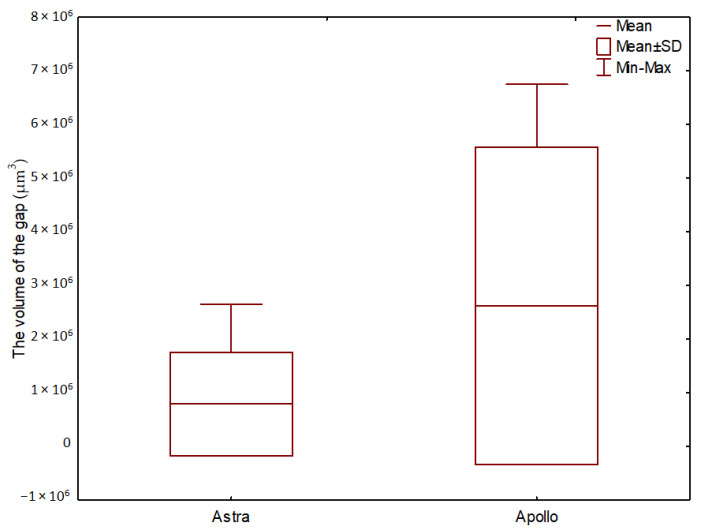
The volume of the microgap for Apollo and Astra.

The average microgap volume was greater for Apollo (0.00262 ± 0.002963 mm^3^) than for Astra (0.000788 ± 0.000967 mm^3^) (Figure 6). Moreover, no statistically significant differences in volumes of microgaps were found between study groups (*p* > 0.05).

Additionally, no statistically significant differences were found between linear (2D) and volumetric (3D) measurements.

## 4. Discussion

The significance of microgaps between implants and abutments seems to be underestimated, even though they may cause severe clinical complications. A microgap creates space for bacteria accumulation, especially when the abutment–implant connection is characterized by micromovements. From the technical point of view, fitting of two different elements results in a microgap between them. Such elements (i.e., implant and abutment) cannot be exactly fitted due to the precision limitation of the manufacturing process. Moreover, as the size of the microgap grows, microleakage and misfit at the abutment–implant connection increase [21]. Excessive bacterial contamination may occur in screw-retained restoration between two fixed structures, namely the implant and the abutment. Bacteria localized at the implant–abutment connection could induce inflammation around the implant because it is located near the alveolar crest and soft tissue. In addition, the longer the interaction of bacteria with bone and gingiva, the greater the risk for bone resorption due to the upregulation of cytokines and osteoclasts (osteoclasttogenesis) [38]. For this reason, excessive bacterial contamination can cause periimplantitis and consequently implant loss [39,40].

In the present study, titanium implants with conical connections were selected because of their claimed good marginal fit and popularity [41,42,43,44]. Moreover, a straight titanium abutment was chosen because of the wide range of modifications that could be suitable for more demanding prosthetic conditions (i.e., single crowns and longer bridges, as well as cemented or screwed restorations). Furthermore, individual, compatible machined abutments from another manufacturer were selected for comparison; it is common that clinicians prefer different types of abutments due to higher availability and lower prices. It is worth emphasizing that these two types of abutments have not been compared until now.

The formation of the implant–abutment microgap could be related to some mechanical properties of the implant–abutment connection, namely type of connection, shape, material used and surface preparation [4]. This microgap can lead to several complications, i.e., implant loss of preload, prosthetic screw fracture, bacterial microleakage and contamination of implant–abutment connection and crown loosening [45].

Two types of abutments are available nowadays, namely custom-made and individually casted. Custom-made abutments showed the best marginal fit in contrast to casted ones due to production (dimension) repeatability [46]. Moreover, the long-term microleakage of Cast On (Straumann, Basel, Switzerland) and Castable (Rhein 83, Bologna, Italy) abutments was comparable, although significantly higher than that of custom-made (pre-machined) ones [5].

The study investigating tapered implant systems with dedicated abutments (Nobel Biocare Replace CC, Ankylos A11, Neodent Drive CM, Conexão Flash) revealed a lack of adaptation at the implant–abutment interface, which caused bacterial microleakage [47]. Additionally, the Morse taper exhibited a better marginal seal than the butt-joint connection thanks to the favourable design of the connection and the implant tapering degree being between 6 and16 degrees [48,49,50]. Interestingly, the size of the microgap for the Morse taper was reported to be up to 0.5–4.6 µm, which could be defined as a very good marginal seal preventing the leakage of most bacterial species (the size of oral cavity bacteria is 0.2–1.5 µm in width and 2–10 µm in length) [13,51]. It is worth emphasizing that friction influences the fixation of taper connection. Therefore, the fitting degree is essentially associated with the connecting area and taper degree. The smaller the taper degree, the larger the removing force and the tighter the connection [52].

The present study demonstrated a very good marginal fit between the implant and the abutment in both the evaluated systems, as most of the specimens did not exhibit any microgaps. The parallelism of abutment to implant led to similar results for all measured positions in both study groups. Except for two Apollo abutments and one Astra abutment, all specimens revealed microgaps (at levels A–D) below 3 µm. Additionally, the size of microgaps at level A and D was higher than at level B and C in both study groups; however, the difference was not statistically significant. This result might imply that the seal between the implant and the abutment was assured by the middle part of this connection. The results of the current study are in agreement with those of Toia et al., who showed good adaptation of an Astra Osseospeed EV with a TiDesign abutment; the average value of microgaps was below 5 µm [53]. It is worth emphasizing that the size of the microgap for Apollo abutments was higher than for Astra ones; however, the difference was not statistically significant. Interestingly, it was found that only the microgap at the midpoint of the implant–abutment interface was significantly smaller for the original than for the non-original abutment [54,55,56]. Moreover, the width of the microgaps often exceeded the size of bacteria [51]. Therefore, it can be assumed that the observed microgaps at implant–abutment connections could result in bacterial microleakage and subsequent contamination of this area [51]. Additionally, the larger the microgap, the higher the risk of micromovement at the interface area. As a consequence, the risk of screw loosening and of potential mechanical complications may increase. According to the literature, the microgap between the implant and the abutment has a large impact on implant treatment success [4,20,51,57,58,59]. However, it should be mentioned that microgaps of 10 µm or less exert no harmful effects, either to hard or to soft tissues [54,55,56].

The application of MCT in the present study also provided the opportunity to measure the volume of microgaps at the interface between the implant and the abutment. The average volume of microgaps was greater for Apollo (0.00262 ± 0.002963 mm^3^) than for Astra (0.000788 ± 0.000967 mm^3^). However, the difference was not statistically significant. Thus, the null hypothesis was accepted. This parameter was also investigated in the literature; one study did not find any difference between the two abutment systems tested [60]. Hence, Cardoso et al. reported that the volume of microgaps amounted from 0.67 ± 0.29 mm^3^ for corresponding abutments mounted on external hexagon implants (S.I.N implants, São Paulo, SP, Brazil) up to 1.42 ± 0.28 mm^3^ for abutments manufactured by another company (Odontofix LTDA, Ribeirão Preto, SP, Brazil) [61]. Other researchers found that stock abutments exhibited significantly higher volume of microgaps than CAD/CAM ones [62]. It is worth emphasizing that the volume of microgaps found in the present study was500–1000 times smaller than in the abovementioned research, which suggests good fit of both abutments tested. Furthermore, the high value for standard deviation implies great variability in the fit between the implant and abutment analysed in the present study. Interestingly, no statistically significant differences were found between linear (2D) and volumetric (3D) measurements. This result was confirmed by Ramalho et al. [60].

The microgap for most connection types ranges between 0.1 and 10.0 µm before loading [52,63]. Similarly to the present study, a conical-hex connection (with 11° angulation) (Astra) was revealed to bethe best fit (the lowest leakage volumewas0.043 µL and the lowest leakage percentage was 1.48%) when compared to Euroteknika (0.09 µL; 6.93%) and Dentium (0.07 µL; 4.6%) [64].

Interestingly, in general, no microgaps were found for the Morse cone (conical connection) (Universal II HI Implacil De Bortoli, Sao Paulo, Brazil) in MCT; gaps (0.3 ± 0.1µm) were present only in a few areas [65]. Corresponding results (MCT) for the microgap (0.26–0.5 µm) were observed for the Morse taper connection by Bagegni et al. [58]. However, higher values of vertical misfit (14.0 ± 2.0 µm) were noted for this connection, but in the internal surface of the implant, no species of microorganisms were found [21]. Measurements (without sectioning of the samples) were performed with a stereomicroscope (80× mag.). It is worth emphasizing that due to the shadow effect, measurement error might occur [21]. In the MCT study, the Morse cone exhibited similar microgap values for the butt-joint connection (0.26–0.47 µm) [58].

Other MCT studies investigating microgaps for internal hexagon connections showed inconsistent results. Namely, some authors indicated a low microgap value of 0.97 µm [25], while others indicated significantly higher ones (6.3 ± 2.5 µm) [65].

Moreover, the vertical misfit of the external hexagon connection varied from 11 up to 14 µm (depending on surface), and consequently, in the microbial leakage study, several microbial species were found to cause periimplantitis [21]. MCT examination also presented microgaps in the external hexagon connection with dimensions up to 1.22 µm [25].

On the other hand, the literature presents inconclusive results regarding size of microgaps after loading (simulating post-load clinical performance) [28,40,51,58,66,67]. It has been advocated that the size of microgaps decreased significantly for internal Morse cone and butt-joint connections compared to internal hexagon connections [58]. The favourable performance of Morse taper over external hexagonal and internal parallel connections was previously reported in other studies [59,68]. According to systematic review, internal hexagon and Morse taper connections showed lower microleakage in dynamic loading conditions when compared to other types of connections [69].

Moreover, vertical misfit for the Morse cone amounted up to 13.7 (±1.1) µm [21] and microgaps of 0.11–0.26 µm (SIC- vantage max^®^, SIC Invent AG, Basel, Switzerland) were determined in the MCT study [58]. Additionally, another research study revealed a microgap of 3.34 ± 2.17 μm for the Morse taper [70]. Furthermore, the microgap significantly decreased after cyclic loading, from 0.7 μm up to 1.35 ± 0.64 μm for the Morse cone [70].

In another study using a scanning laser microscope (SLM), the mean microgap of all measured implant–abutment interfaces amounted up to 2.3–5.6 µm for internal hexagon connections (Osseotite Certain, Nobel Biocare) and tube-in-tube connections (Camlog, Artatec) [28]. 

Moreover, a microgap of 0.3–12 µm for internal conical connections was detected by Blum et al. by means of synchrotron-based microtomography [66]. On the other hand, others reported a significant increase in the microgap size after cyclic loading in internal conical connections (Nobel Active, Ankylos, Astra Osseospeed TX, Straumann Bone Level) [66]. Interestingly, a higher microgap size (4.9 ± 2.1) µm was found for internal connections (tube-in-tube) (CAMLOG) using SLM [28].

Additionally, for external hexagon connections, amicrogap of 13.8 (±2.0) µm was found using a stereomicroscope (80× magnification) [21]. It is worth mentioning that the microleakage decreased when the torque recommended by the manufacturer (30 N·cm) was applied [40,67]. On the contrary, significantly lower microgap sizes for external hexagon connections (OSSEOTITE External Hex—2.8 ± 0.72 µm and Brånemark System MKIII—2.3 ± 0.49 µm) were detected using SLM [28].

Moreover, a MCT study of butt-joint connections (SICmax^®^, SIC Invent AG, Basel, Switzerland) revealed microgaps of 0.21–0.25 µm [58].

In a SLM study, the microgap and horizontal discrepancy for internal hexagon connections (OSSEOTITE Certain) amounted up to 3.2 (±0.97) µm and 7.6–16.0 µm, respectively [28].

To conclude, based on the current literature, the evaluation of microgaps before and after cyclic loading revealed different results for various types of implant connections [21,28,40,58,67,69]. In general, the highest adaptation of the implant–abutment interface was found for the conical connection [58,59,66,68]. Indeed, the size of microgaps increased significantly after loading [21,28,40,66,67,69]. However, some studies showed significant decreases in microgap size for the Morse cone [58,70] and butt-joint connections [58] after loading. Moreover, inconsistent results were found for external hexagon connections [21,71,72,73]. Some researchers indicated no changes in misfit of implant components [54,72], while others reported increased [73] or reduced [74] misfit after loading. This diversity of results may be explained by different experimental models, namely number of implants restored and loaded, value and direction of loading forces and abutment characteristics (alloy, casting procedure) [21,71,72,73]. Moreover, the joint stability against the functional load can be affected by abutment sinking, surface sinking of the implant system, abutment screw deformation and joint micromovement [75].

Furthermore, some differences in misfit were found with regard to material and the surface processing method applied. Ti abutments showed a 3–7 times better marginal fit than different zirconium oxide abutments [43]. On the other hand, for horizontal microgaps, the highest misfit results were observed for machined Ti abutments versus pre-machined cast-on palladium abutments, plastic burnout abutments cast with nickel chromium alloy and plastic burnout abutments cast with cobalt chromium alloy [32]. Moreover, another study revealed a significantly better marginal fit for internal conical connections of titanium (2.0–6.6 µm) than of zirconium abutment (7.4–26.7 µm) [75].

Some limitations of the present study should be recognized. First of all, the variability of the obtained data and relatively high value of standard deviation might be due to the limited sample size or the non-standardized size of abutments. Thus, studies using a larger sample size should be performed in the future. Additionally, only two abutments attached to one implant were tested in the study. Therefore, other abutments (including chairside CAD-CAM) and implants and their combinations should be investigated. The microgap at the abutment–implant interface before and after load should also be measured. Additionally, microgaps were measured by means of only one method, namely, MCT; thus, other investigation methods should be employed, including SEM observations, to provide a wider perspective on this issue. Subsequently, the design of this laboratory study does not reflect oral cavity conditions, namely occlusal loads and function, plaque and microorganism retention, saliva, pH and temperature changes, gingiva and bone anatomy. Therefore, clinical studies should be performed to complement these results.

## 5. Conclusions

It can be concluded that most samples did not exhibit any microgaps. Furthermore, the linear and volumetric dimensions of microgaps observed at the interface between Apollo or Astra abutments and Astra implants were comparable. Additionally, all tested components presented microgaps (if any) that were clinically acceptable. However, the microgap size of the Apollo abutment was higher and more variable than that of the Astra one.

The findings obtained in the present study may be of clinical relevance, in that implant–abutment misfit is known to cause subsequent microleakage and mechanical damage. As a consequence of bacteria colonization, inflammation around the implant may develop. Additionally, increased mechanical stress on the implant–abutment connection and surrounding bone tissue might result in further destruction of these structures. Therefore, an implant system with high quality of fit between components should be applied to restore missing teeth.

## Figures and Tables

**Table 1:** Study groups.

Astra		Apollo	
Implant	Abutment	Implant	Abutment
OsseoSpeed EV 4.2 mm x 11 mm (Astra Tech, Dentsply, York, PA, USA) 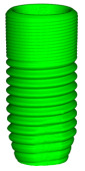	TiDesign EV (Astra Tech, Dentsply, York, PA, USA) 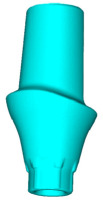	OsseoSpeed EV 4.2 mm x 11 mm (Astra Tech, Dentsply, York, PA, USA) 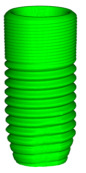	Individual Titanium Abutment (Apollo Implants Components, Pabianice, Poland) 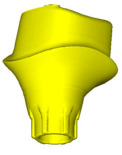

## Data Availability

Not applicable.

## Appendix A

**Table A1:** Presence (+) or absence (−) of microgaps in different areas of the implant–abutment interface.

Study Group	Sample	Measurement Site			
		A	B	C	D
Astra	1	−	−	−	−
	2	−	−	−	−
	3	−	−	−	−
	4	+	−	−	+
	5	−	−	−	−
	6	−	−	−	−
	7	−	−	−	−
	8	−	−	−	−
	9	−	−	−	−
	10	−	−	−	+
Apollo	1	−	−	−	−
	2	+	−	−	+
	3	−	−	−	−
	4	−	−	−	−
	5	+	+	+	+
	6	−	−	−	−
	7	−	−	−	−
	8	−	−	−	+
	9	−	−	−	−
	10	−	−	−	−
